# Centromeres under Pressure: Evolutionary Innovation in Conflict with Conserved Function

**DOI:** 10.3390/genes11080912

**Published:** 2020-08-10

**Authors:** Elisa Balzano, Simona Giunta

**Affiliations:** 1 Dipartimento di Biologia e Biotecnologie “Charles Darwin”, Sapienza Università di Roma, 00185 Roma, Italy; elisa.balzano@uniroma1.it; 2 Laboratory of Chromosome and Cell Biology, The Rockefeller University, 1230 York Avenue, New York, NY 10065, USA

**Keywords:** centromere, repetitive DNA, mutagenesis, centromere evolution, HORs, chromosome instability

## Abstract

Centromeres are essential genetic elements that enable spindle microtubule attachment for chromosome segregation during mitosis and meiosis. While this function is preserved across species, centromeres display an array of dynamic features, including: (1) rapidly evolving DNA; (2) wide evolutionary diversity in size, shape and organization; (3) evidence of mutational processes to generate homogenized repetitive arrays that characterize centromeres in several species; (4) tolerance to changes in position, as in the case of neocentromeres; and (5) intrinsic fragility derived by sequence composition and secondary DNA structures. Centromere drive underlies rapid centromere DNA evolution due to the “selfish” pursuit to bias meiotic transmission and promote the propagation of stronger centromeres. Yet, the origins of other dynamic features of centromeres remain unclear. Here, we review our current understanding of centromere evolution and plasticity. We also detail the mutagenic processes proposed to shape the divergent genetic nature of centromeres. Changes to centromeres are not simply evolutionary relics, but ongoing shifts that on one side promote centromere flexibility, but on the other can undermine centromere integrity and function with potential pathological implications such as genome instability.

## 1. An Introduction to Centromere Diversity

In 1882, Walter Flemming observed the central structure that forms the primary constriction on mitotic chromosomes [1], later named the centromere [2]. Despite its early cytological discovery, the centromere remains a fascinating and rather mysterious region of the genome. A hundred years after Flemming’s observation, the smallest centromere, suitably named “point centromere”, was characterized by Louise Clarke and John Carbon in the budding yeast *Saccharomyces cerevisiae* [3], made of a single centromere-specific nucleosome [4]. Already from these early studies, two key and apparently contrasting aspects of centromere biology emerged: great heterogeneity in centromere DNA size, organization and structure across species [5,6], while holding an essential and evolutionarily conserved function in enabling chromosome segregation [7]. Centromeres can be broadly classified into different types (Table 1) based on relative size: (1) point centromeres, which are rare and only found in fungi; (2) regional centromeres, which are the most common type of centromere where a specific genomic region defines the centromere location (because regional centromeres can vary widely in size, a further sub-classification has been proposed between short (<40 kb) and long (>40 kb) regional centromeres [8]); (3) holocentric centromeres, which are diffused and encompass the entire chromosome (recently, single base pair resolution data have shown that holocentric organisms like *C. elegans* in reality consist of hundreds of budding yeast-like point centromeres in a “polycentric” set up); and (4) meta-polycentric centromeres, which are a recently-added, rare category where the centromeres are alternated and thus extended to cover a section of the chromosome. These categories that highlight the genetic diversity of centromeres are recapitulated in Table 1, and described in detail below.

A unified consensus for the centromere can be reached when describing its conserved and essential role: centromeres are necessary for the correct inheritance of genetic material by enabling chromosome attachment to the spindle microtubules during each round of cell division [22,23]. Centromeres as *conditio sine qua non* for genome inheritance are highlighted by the quest to engineer human artificial chromosomes (HACs). HACs require centromeric DNA, or centromere chromatin, in order to be stably transmitted over cellular generations [24].

Centromere specialization is primarily determined by a unique chromatin environment founded on the presence of a centromere-specific nucleosome containing the histone H3 variant protein centromere protein A (CENP-A) that serves as a docking template for centromere factor binding and mitotic kinetochore assembly, and epigenetically encodes the transgenerational inheritance and propagation of the centromeric locus [25]. Underscoring its essential and evolutionarily conserved function, homologs for CENP-A are found in many species throughout evolution and are studied in a variety of laboratory model organisms (Table 2) [26,27].

**Figure 1:**
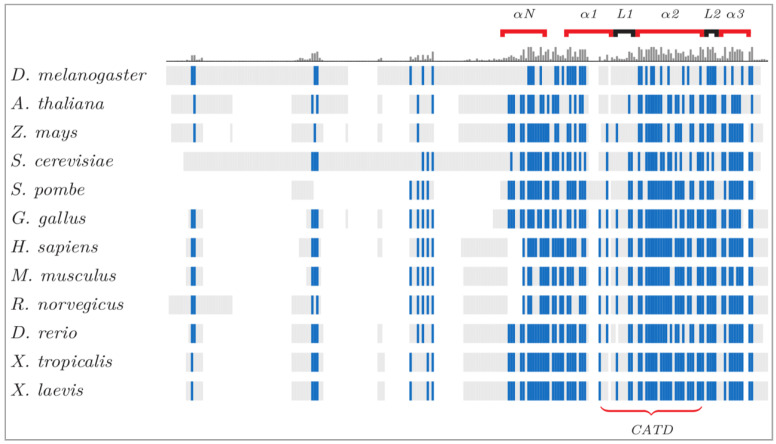
CenH3 protein alignments, conservation and diversity across species. The structural elements of CenH3 proteins are illustrated, with conserved residues in blue. The histogram above the sequences shows the conserved regions: the carboxyl terminal domain and its components (L1 and α-helix) are highly preserved across eukaryotes. The shared CENP-A Targeting Domain (CATD) drives the association between proteins and centromeres [50]. Despite the variability of the amino terminal tail, this domain contains a phosphorylatable serine for CenH3 mitotic function [51]. This image is courtesy of Damien Goutte-Gattat [52].

The centromere histone is preserved in flies as the centromere identifier (Cid) [32,43], in worms as histone H3-like centromeric protein (HCP-3) [36], in plants and fungi as centromeric histone 3 (CenH3), in fission yeast as centromere-specific histone H3 (Cnp1) [30], in budding yeast as chromosome segregation 4 (Cse4) [28], in mouse as (Cenp-a) [38] and in human as CENP-A [40,41], as well as other species (Table 2). The ubiquity and conservation of the centromere-specific histone variant prompted the suggestion for a common designation of CenH3/CENP-A [44]. As more model organisms are being studied, our understanding of centromere epigenetic specification and its diversity broadens. Recent work in the garden pea *Pisum sativum* show that it contains multiple copies of CenH3 protein to generate an extended primary constriction, defined as a “meta-polycentric centromere” with alternated CenH3 domains [16,45]. Similar scattered features are also seen in other organisms [46]. CENP-A is interspersed with the canonical H3-containing nucleosome in a way that is conserved from flies to humans [46], and forms high-density islands/sub-domains of CENP-A across the human centromeres, as it was reported looking at stretched chromatin fibers [47]. The expansion of the peculiar centromere structure of *Pisum* was also found in another legume tribe species, *Lathyrus*, where it has an additional copy of the CENH3 gene that was not seen in other phylogenetically-related species [48], underscoring centromere genetic and epigenetic diversity across even closely related species. While a structural relationship exists between human centromere proteins that mark the functional “centrochromatin” [49], CENP-A and other centromere proteins are amongst the fastest changing during evolution, with hyper-variable regions, divergence in length (Table 2) and divergence in overall sequence and domain composition (Figure 1).

Intriguingly, new evidence has demonstrated the absence of the largely conserved centromere histone in some organisms. CenH3-independent centromeres were found in the African sleeping sickness parasite *Trypanosoma brucei* [53] and in four lineages of insects, underscoring an ancient transition associated with a switch from regional or point centromeres to holocentric centromeres that was accompanied by loss of the centromere-specific histone [54]. This raises the question as to why some holocentric organisms retain a centromere-specific histone while others do not. Partly, it may relate to the conservation of kinetochore proteins present among holocentric and monocentric centromeres even in species where CenH3 is lost [55]. Retaining kinetochore assembly is the ultimate goal to enable centromere activity [56]. In the case of specific insect lineages, the holocentric centromeres devoid of CenH3 still present canonical kinetochore proteins, especially the outer part where the kinetochore interfaces with microtubules [54,57]. *Trypanosoma brucei* remains to date as an exception, showcasing extremely divergent outer kinetochore components defined as an “unconventional” kinetochore which is made up of 20 apomorphic kinetoplastid kinetochore proteins (KKT1–20) not conserved across the other flagellated members of the monophyletic group of Euglenozoa [53,58]. The *Trypanosoma* “exception” challenges the assumption that centromere function is funded on its epigenetic specification. Other systems may exist where chromosome segregation is free from the imposed presence of CenH3, or even “canonical” kinetochore constrains [59]. Further investigations into CenH3 divergent evolution, holocentromere condition and cases that lack epigenetic specification for centromeres will shed light on essential and universal requirements for chromosome segregation.

The wide diversity of centromere proteinaceous constituents is paralleled by the progressive mutability of underlying centromere DNA [60]. At the genetic level, centromere sequences are characterized by repetitive DNA, often rich in A/T nucleotides and arranged in tandem units as found in many organisms. The high representation of repeats across species implies a bias for reiterated DNA in supporting centromere formation and function [61]. Yet the finding by Voullaire et al. (1993) of an ectopic human centromere, so-called neocentromere, on marker chromosomes 10 deprived of repetitive DNA brought the requirements for DNA repeats at centromere under scrutiny [62]. Neocentromeres seem to have a sequence-independent formation [63], underscoring the epigenetic foundation of centromeres [64,65,66]. Alphoid-less centromeres likely originated from neocentromeres. An absence of satellite repeats was seen in the horse centromere on chromosome 11 (Equus Caballus 11, ECA11) [67], in zebra for chromosomes 2, 5, 7, 13, 18–21 [68] and in the donkey centromeres 11 and 16 [69]. These satellite-free centromeres form primary constrictions and still guarantee segregation fidelity [70]. In particular, ECA11 is well conserved in the syntenic region in other mammals and its two internal regions of 136 and 99 kb both bind CENP-A and CENP-B [71], respectively, suggesting robust propagation even in the absence of satellite DNA repeats.

A reconciliation regarding the functionality of repetitive centromere sequences was offered by recent data pointing to a role for CENP-B in fulfilling centromere specification by stabilizing and partly recruiting CENP-C directly to the centromere in human cells depleted of CENP-A [72,73]. CENP-B is recruited to a specific consensus sequence, the CENP-B box present within human α-satellite repeats [74]. Thus, CENP-B-containing centromeres are specified by a concerted contribution of both CENP-A loading, in a sequence independent manner, and of CENP-B recruitment to the CENP-B box [75]. So, while epigenetically CENP-A is necessary and sufficient to establish a centromere in proliferating somatic cells [76], whether it is on a HAC [24] in an ectopic location [63,77] or on a lactose operon (LacO) array [78], recent evidence shows that CENP-B may be able to fully compensate for CENP-A in enabling centromere specification, formation, positioning and transgenerational inheritance [72,73] (Daniele Fachinetti and Sebastian Hoffman; personal communication).

*Cis*-acting α-satellite sequences are not sufficient to define a functional centromere. Indeed, “non-alphoid centromeres” have been found in plants [79,80], in birds [81], among Equidae subspecies (e.g., speciation between horse and donkey) [69,82,83], in different primate species [84] and in humans [85]. This means that new centromere sites are generated without a corresponding alteration in DNA organization and they are still undergoing repositioning. Indeed, new centromere formation could represent a way to insert inter- and intra-species diversity [86,87,88].

Ectopic centromere formation represents an opportunity to re-localize the centromere to a new position outside the endogenous site, giving rise to a functional neocentromere which enables cell division upon disruption of the endogenous centromere. The configuration of the neocentromere can occur at a distance from the endogenous centromere, as found within inverted duplications between a breakpoint and a telomere end [89]. The ability for kinetochore protein assembly on the new locus is assisted by CENP-A recruitment to the neocentromere [90]. Interestingly, chromosomes containing active neocentromeres can be maintained over generations, implying that the chromosomal positioning of the centromere region retains flexibility in its localization and can promote sister chromatid separation even when decentered or greatly shifted from the endogenous locus. Thus, the pliability in accommodating centromere functionality over diverse sequences and variable overall size also extends to adaptability to different locations along the chromosome [91]. Similarly to gene duplication being the first step toward divergence and functional innovation, the establishment of a new, competent centromere site outside of the endogenous locus offers flexibility and sustained functionality. Amongst the many plausible mechanisms for neocentromere formation, the recently reported ectopic CENP-A loading [92] and/or binding transiently to DNA double strand breaks (DSBs) [93] may represent favorable sites for the initiation of neocentromere formation, the establishment of a functional de novo centromere [94,95,96,97] and for its stabilization during subsequent generations [98]. Leo et al. offers a detailed review in this *Genes* Centromere Stability special issue of the different models of neocentromere formation [99].

Following the evolutionary footsteps of centromere sequences and proteins can help unravel some of the aforementioned riddles and paradoxes in centromere biology. Here, we have delved into the conflict between evolutionarily and ongoing mutagenesis in centromere DNA and whether these processes may impact the conserved and essential functions of centromeres. How these seemingly detrimental mechanisms converge to undermine centromere function while also being important contributors to centromere biology and evolution will be discussed (Section 2).

## 2. Centromere Organizational Diversity in Light of Evolution

From the smallest and simplest centromere of *Saccharomyces cerevisiae* to the large and complex ones found in higher eukaryotes, including human megabase-sized ones, the evolutionary compulsion to sustain variability in order to exploit this locus for chromosome segregation is evident [100].

A case in point is the fast evolving “point” centromere of budding yeast *S. cerevisiae* with as little as ~125 bp (base pair) consensus AT-rich sequences [4,28,101].

**Figure 2:**
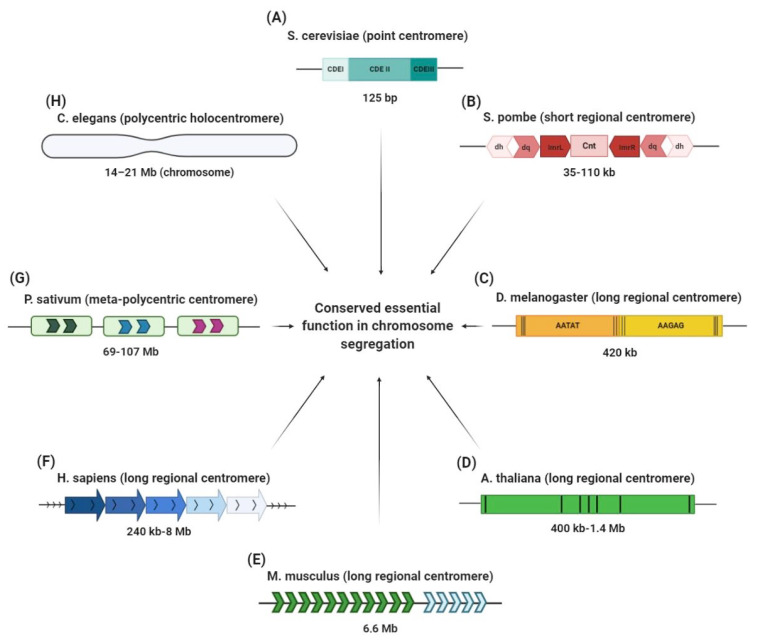
Centromere structures in different eukaryotes. (**A**) The S. cerevisiae point centromere is 125 bp in size and it is composed of three centromere DNA elements (CDEs): CDEI, CDEII and CDEIII. (**B**) The S. pombe centromere is made of inner (ImrL and ImrR) and outer (dg and dh) inverted repetitive sequences that flank a central unique sequence (Cnt). (**C**) The two main satellite domains (AATAT and AAGAG) of the D. melanogaster centromere are interspersed with transposable elements (black lines). (**D**) A. thaliana has a 180 bp repeat unit intermingled with retrotransposons (black lines). (**E**) The mouse centromere is made up of major satellite sequences (MaSat) of 234 bp monomers (spanning ~6 Mb; green arrows) and minor satellite sequences (MiSat) of 120 bp monomers (spanning ~600 kb; blue arrows). (**F**) Human centromeres contain tandem repeats of α-satellite 171 bp monomers organized head to tail into higher order repeats (HORs). (**G**) The meta-polycentric centromere of P. sativum is a very long centromere of 13 families of satellite DNA repeats and one family of Ty3/gypsy retrotransposons, organized into 3–5 domains containing CenH3. (**H**) The polycentric or holocentric centromere of C. elegans covers the entire length of chromosome on which there are several points for microtubule attachment. In spite of this great diversity, all these centromeres perform faithful roles in chromosome segregation.

Each centromere has three centromere DNA elements (centromere determining elements, CDEs) for the association of the centromere DNA binding protein complex: CDEI (~8 bp), CDEII (~78–86 bp) and CDEIII (~25 bp) (Figure 2A) [102,103,104,105,106]. Cse4 maps on the CDEII DNA element and forms a modified histone octamer, with different studies proposing a variety of models for this nucleosome:homotypic tetrasome (Cse4/H4)2 [107], hexasomes with non-histone proteins (Cse4-H4/Scm3)2 [108], asymmetric/mixed octasomes (Cse4/H3/(H4/H2B/H2A) [109] and single right-handed hemisomes (CenH3/H4/H2A/H2B) wrapping the ~80 bp of DNA centromeric sequence [110,111].

In fission yeast Schizosaccharomyces pombe, the centromeric region is large relative to the total genome size, spanning 35–110 kb, of which ~4 kb represents a unique central sequence (cnt) flanked by two inverted repetitive sequences (ImrL and ImrR) (Figure 2B) [10,112].

Next based on overall size is the 420 kb repetitive centromere of *Drosophila melanogaster*, composed of over 85% satellite DNA interrupted by the presence of transposable elements (TE) (Figure 2C) [113].

A very similar composition of satellite DNA and centromeric transposable elements was also found in plants, such as *Arabidopsis thaliana* [114], *Oryza sativa* [115] and *Zea mays* [13]. Elements of diversity in these plant satellite DNA are displayed by the size of the basic unit present and number of reiterations of these units which make up the centromeres, ranging from 400 kb to 1.4 Mb. For instance, the *Arabidopsis* centromere has a 180 bp monomer (Figure 2D) [11,116], rice has a 155 bp satellite CentO unit [12] and maize contains a 156 bp satellite unit named CentC [13]. These repeated units, while divergent, all specifically bind well-characterized centromere proteins. Satellite sequences found in the mouse centromere also contain repetitive domains with distinct unit sizes [15,117]. The mouse centromere is organized into minor satellite DNA with a 120 bp homogenized unit that constitutes the core centromere region, and flanking major satellite DNA of pericentromeric heterochromatin that is made up of less-ordered 234 bp units (Figure 2E) [15,118]. In humans, the centromere is also distinct from the flanking pericentromere. The former is made up of tandemly organized repeats, called α-satellite DNA, while the latter is made of monomeric α-satellite units and other types of repeats. Within the core centromere, the 171 bp monomeric units of α-satellite DNA arranged in tandem share between 50% to 70% sequence homology. Several repeat units form a higher order repeat (HOR) block that is reiterated with a similarity of 97–100% to make up a homogenized array spanning several megabases, usually 2–5 Mb (Figure 2F) [16,61]. Notably, each human chromosome has a different number of monomers that make up its HOR, with some chromosome-specific sequences contained within the homogenized array. Thus, sequence diversity is not only found across species but also within species, across the karyotype.

In addition to the aforementioned regional centromeres, large or small (which we categorized as short and long regional centromeres, as in Table 1), there are other kinds of centromere genetic structures with less common organization, including organisms that have multiple or diffused centromeres. A striking example of a centromere which is an intermediate between a monocentric (single) centromere, and a polycentric, is the garden pea, *P. sativum*. Similarly to other species equipped with satellite DNA, the *P. sativum* centromere is constructed on tandem repeated domains of 13 individual families of satellite DNA and one family of Ty3/gypsy retrotransposons (Figure 2G). The *Pisum* meta-polycentric centromere is then made up of 1–5 domains. Reminiscent of the multiple centromeric arrays found in human chromosomes, only one array represents the active centromere that forms the kinetochore. Notably, the garden peas’ centromere is considered polycentric because multiple active arrays contribute to a linear-like kinetochore [17], unlike other centromeres where only one of the repetitive arrays is functional [119].

In addition to the monocentromere and meta-polycentric centromere described above with a defined site for each chromosome, the holocentromere is dispersed to the total length of chromosome with a non-localized kinetochore. The holocentric condition is spread in several phyla, implying multiple distinct and independent occurrences during evolution [120]. The *Caenorhabditis elegans* centromere is a prime example of a holocentric organism, where the centromere encompasses the full length of the chromosome (14–21 Mb) (Figure 2H) [21], yet it is still dependent on the H3-like centromere histone HCP-3 for chromosome segregation during mitosis [36,121]. Through the evolutionary lens, centromere organization looks somewhat stochastic, with different species having evolved their own particular way to adapt a centromere locus for chromosome segregation. Importantly, while centromeres can exist in different forms and arrangements, their purpose to achieve accurate division of genetic material is always accomplished [22,122].

Indeed, primary constriction size appears invariant and with a constant scale of magnitude from yeast to human [123]. Thus, despite the great evolutionary diversity and organization across eukaryotes, centromere function in chromosome segregation remains conserved.

### Centromere Drive: From Conflicts to Benefits

A rapid and heterogeneous evolution of centromere components across eukaryotes is in disagreement with its vital and conserved centromere function [7,124]. Yet, these mutagenic changes must be in accord with a synchronized shift of centromeric elements that provide an evolutionary advantage. A plausible reason for this fast centromere evolution–adaptation paradox is elegantly provided by the “centromere drive” hypothesis formulated by Malik and Henikoff [7,125], where centromere DNA and protein components co-evolve under genetic conflict [126]. Centromere drive sees centromeres not only as essential regions of the genome during cell division, but also as “selfish genetic elements” that have an opportunity to play tug-of-war during the first asymmetric division (MI) in female meiosis and bias their transmission [126,127]. In fact, in the centromere drive model, the stronger centromeres segregate successfully with respect to the competitors. Their ability to exploit the asymmetry of oocyte meiosis, overthrowing Mendelian genetic laws [127], means that there is a Darwinian selection between centromeric variants for their transmission to the gametes and consequently for their inheritance, which underlies the constant genetic changes as a continued quest toward improved strength and favored inheritance. There are several examples demonstrating the validity of the centromere drive hypothesis. Recent elegant proofs were provided by the Lampson lab using crosses between mouse strains with different amounts of centromere proteins. The “stronger” centromere was preferentially inherited during female meiosis due to increased levels of kinetochore proteins contributing to the likelihood of transmission to the egg [128]. The presence of mutational changes in centromeric sequences is reconciled with simultaneous conformational changes in centromeric proteins, generating more microtubule attachment sites [129,130,131]. Lampson and collaborators set up a system to investigate the implication of changes in satellite DNA in recruiting the kinetochore complex. They found a 6–10-fold increment of minor satellite mouse centromeric repeats in “strong” centromeres compared to the “weaker” centromere mouse strain [129,132]. The size difference translates into increased retention of CENP-B protein on its DNA binding motifs, CENP-B box present on the minor satellite that consequentially recruits additional CENP-A proteins [133] and, in turn, is responsible for the robust assembly of the outer kinetochore for robust attachment to the asymmetric meiotic spindle [129]. The stronger centromeres are able to orient towards the egg pole and remain in the mature oocyte, winning a spot in self-propagation [128,133]. In addition to centromere DNA changes, meiosis can also be biased by other features, including spindle asymmetries [128].

Even though this evidence elucidates the advantage of centromere evolutionary changes, deleterious effects must also be taken into consideration, including unbalanced segregation that could generate incompatible post-zygotic hybrids contributing to speciation [124,134].

Centromere rearrangements are protagonists in karyotypic divergence, as in the case of the horse and donkey. Changes in centromere repositioning created chromosomal structural variations that act like a “genetic barrier” between these two species due to the odd rate of meiotic chromosome recombination, which causes the gametogenic failure in mules [135].

To contrast this constraint, CenH3 gene duplications are positively evolving, with the vast majority becoming pseudogenes and fixing in the population as they are able to adapt to the selection imposed by changes in centromeric sequences [136]. For instance, *Mimulus aurantiacus* displays many CenH3 duplication events under a divergent process in which paralogs differentiate with distinct sub-specialized functions [136]. CenH3 duplication and divergence are also seen in *Drosophila* where five duplications of the Cid gene correlate with tissue-specific expression [60,137,138].

Thus, similarly to other evolutionary changes, centromere DNA and centromeric genes use duplications as a mechanism to mitigate rapid mutagenesis. Notably, this rapid evolution of centromere sequences and/or proteins is an irreversible process and on some occasions, it might turn into chromosomal instability [139].

In addition to the issue of speciation, changes at the centromere are not simply evolutionary relics that are now settled, but ongoing shifts in the context of centromere drive. Centromeres may be unstable regions of the genome not just on an evolutionary timescale, but also within the cellular lifetime [140]. Indeed, recombination and rearrangements were found to happen within a single cell cycle in human primary epithelial cells [141]. In Section 3, we will review the mutagenic processes that occurred to form the peculiar genetic structures of centromeres during evolution, and that may continue to undermine centromere stability during cell division.

## 3. Mapping Mutagenic Mechanisms by Following Their Evolutionary Footsteps on Centromere DNA

Centromere DNA is one of the fastest evolving sequences found within the eukaryotic genome. The repetitive nature of centromeres, often in head-to-tail orientation, implies that the repeat units were subjected to expansion and reiteration, followed by other rounds of mutagenesis, to enable formation of the region as we observe it today. To reconstruct the repetitive array, several simulations have been proposed to understand how mutagenesis acts on centromeres to shape their genetic structure. Recombination at the centromere seems obvious yet has remained counter-intuitive. Starting 80 years ago, numerous evidence has been accumulating, demonstrating the negative effects of meiotic recombination within the centromere region [142,143] in different organisms [144]. A reduced level of recombination events at centromeric and immediately flanking sequences during meiosis has long been established, giving a reputation to centromeres as “cold” spots to recombination, as described by Andy Choo, who asked the question: “Why is the centromere so cold (to recombination)?” [145]. Highly condensed chromatin has been thought to repress recombination in order to avoid instability within centromere DNA repeats [146], as well as DNA methylation [147]. Extreme linkage disequilibrium for single nucleotide polymorphisms (SNPs) found at centromeres is another indicator of a low rate of recombination and crossing over events [148,149,150]. Yet, centromere DNA structure and the high degree of homology between satellites across chromosomes are strongly indicative of recombination-driven homogenization and evolution. In addition to evolutionary processes, recombination has been shown to happen to centromeres at relatively high levels during a single cellular generation, with specific factors contributing to its (at least partial) suppression [141,147]. Sister chromatid exchanges were detected in mouse [147] and in human cells [141] using a technique called Centromere-Chromosome Orientation-Fluorescent in situ Hybridization (Cen-CO-FISH) [151], and centromere proteins including human CENP-A contribute to repressing centromere rearrangements [141]. Intriguingly, recombination and other mutagenic processes may be promoted by intrinsic features of centromere repetitive DNA. Given the exceptional flexibility of centromeric repeats, altered topological conformation and secondary structures are likely to occur [142,152,153]. Emerging roles for centromere chromatin in mitigating centromere instability by reducing recombination [141], transposition events and possibly suppressing DNA damage formation indicates an interesting balance between intrinsic or programmed mutagenesis and epigenetic stabilization at centromere.

**Figure 3:**
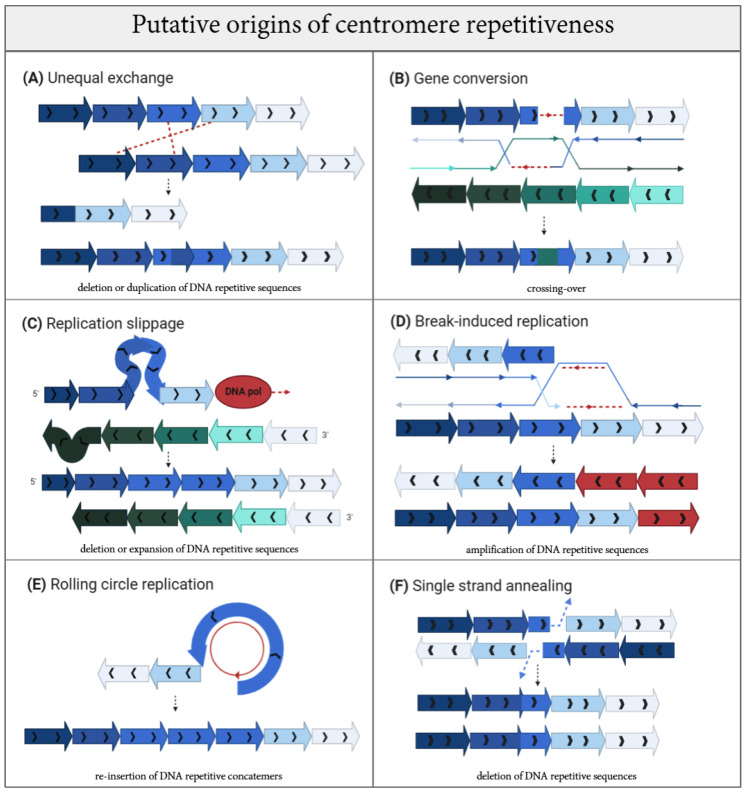
Mutagenic processes that may operate at centromere sequences and have contributed to their repetitive origins. (**A**) Unequal exchange following recombination can cause gain or loss of tandem repeats and DNA rearrangements. (**B**) Gene conversion causes the unidirectional transfer of genetic information among homologous repetitive DNA sequences and can result in reciprocal or non-reciprocal exchange (the latter is depicted). (**C**) Replication slippage on misalignment repeated DNA strands during replication is thought to induce centromere expansion or contraction depending on whether the hairpin (depicted)/distortion is found on the newly synthesized strand (blue repeats) or the bulge (depicted)/distortion is on the template DNA (green repeats). (**D**) Break-induced replication (BIR) repairs one-ended double-stranded break (DSB) substrate, produced by replication fork collapse. (**E**) Rolling circle replication occurs when the 3′ end circularizes, and its replication produces repeated concatemers. (**F**) Single strand annealing (SSA) repairs DSBs through the annealing of complementary ssDNA strands succeeded by DNA tail end digestion and ligation. These repair pathways are essential for maintaining genome stability, yet when operating on repetitive sequences (especially arranged in tandem and sharing high degree of sequence homology like at the centromere), they may result in mutagenic variability as a way for ongoing DNA evolution and shaping.

On an evolutionary timescale, homogenization of centromeric repeats has been speculated to emerge precisely through short and long-range stochastic unequal exchange (Figure 3A) between sister chromatids. These were described in the Smith model [154] by a non-reciprocal recombination between homologous sequences that are neutral to selection [155,156,157]. Similarly, the mechanism of gene conversion (GC) (Figure 3B) [158] is a unidirectional transfer of genetic information from an intact to a broken strand, and can readily account for centromere expansion driven by DNA damage. Depending on the length of GC tracts, they can be called short-tract gene conversions (STGC) for DNA segments ranging between 50 to 200 bp [159,160] or long-tract gene conversions (LTGC) for segments over 1 kb [161,162], with LTGC likely playing a role at large centromeres.

Generally, homology tracts are templates for the resolution of double Holliday junctions (HJ) and synthesis-dependent strand annealing (SDSA) during gene conversion. Both these intermediates are implicated as down-stream processing for the resolution of DNA double stranded breaks (DSB) through DNA damage repair (DDR) pathways. The origins of DSBs within centromere repeats remain unknown. We speculate that stochastic damage can be exacerbated by the intrinsic fragility of centromeres [140]. Another interesting source of DNA damage is represented by transposons. The occurrence of non-allelic gene conversion between duplicated TEs has been demonstrated [163,164] and, while CENP-A nucleosomes seem to play a role in suppressing these TE-mediated mutagenic events, they are thought to retain an active role that impacts the centromere genomic landscape [165,166]. The insertion of TEs and post-insertion events are thought to produce the homogenization of arrays seen among non-homologous chromosomes within the same cell [165]. Indeed, recent evidence in *Monopterus albus* show that two TEs, called GYPSY5-ZM_I retrotransposable element of *Zea mays* and MuDR-13_VV DNA transposable element of *Vitis vinifera*, gave rise to the *Monopterus albus satDNA* repeats MALREP (MALREP-A, MALREP-B, and MALREP-C) through unequal crossing-over [167]. The same mechanism was previously observed in the *P. sativum* tandem repeat satellite PisTR-A, in which the long terminal repeats (LTRs) of the Ty3/gypsy Ogre retrotransposons represent the template for the amplification of satDNA arrays [168] and, thus, contribute to the origin of species-specific centromeric satellites [48,169]. Generation of a new centromere site has also been correlated with the pervasive transcription of TEs that recruit CENP-A through small RNAs called centromere repeat-associated short interacting RNAs (crasiRNAs) [166,169]. Given the recently appreciated role of centromere transcripts and transcription in centromere function [170], it is possible that TEs operate by inducing breaks and/or by exerting the induction of transcription, and both these processes may converge to promote centromere formation. 

High prevalence of gene conversion events are overrepresented in palindromic and reversed repetitive sequences [164]. DNA palindromes appear to be a feature of centromeres and pericentromeres in different species [171,172,173]. Palindromes also have the intrinsic potential to adopt non-canonical B-DNA helix conformations, including Z-DNA, triplex, quadruplex, cruciform [174], again suggesting a multi-step challenge associated with DNA-based transactions like replication, transcription and repair processes at centromere repeats [175]. In addition to palindromes, there are a multitude of alternative DNA secondary structures that centromere repetitive DNA assume, including non-B-DNA [153], triples and G-quadruplex (G4) [176,177,178], i-motifs [179,180], hairpins [181] and loops found at human α-satellites [152]. These and other possible arrangements for three-dimensional DNA folding are expected to directly hinder the replication process as physical barriers. These impediments can also lead to the lower affinity of DNA polymerase for the newly synthetized strand, causing out of register “replication slippage” (Figure 3C) [182]. Replication slippage has been speculated to contribute to centromere repeat amplification, and can provoke either replication fork stalling or collapse, generating a DSB and further promoting mutagenesis [183,184]. DSBs can be repaired through different pathways with specialized protein cascades and diverse outcomes. While DSB repair pathways have been extensively detailed, information on centromeric DSB repair is still lacking. Generally, non-homologous end joining (NHEJ) is a primary pathway of repair utilized throughout the cell cycle that promotes the rapid re-ligation of broken DNA ends without requiring extensive processing. NHEJ is comprised of canonical-NHEJ (c-NHEJ) or alternative-NHEJ (a-NHEJ). The latter can utilize micro-homology between the two broken ends for alignment between sequences of 1–16 nucleotides before rejoining [185]. NHEJ represents an error–prone repair solution, which leaves behind a mutational scar, but such a signature is not obviously observed within the available centromere sequences. Only once a homologous sequence is available after replication can the damaged locus be repaired by homologous recombination (HR). In S-phase and G2, approximately half of all DSBs become substrates for HR using the sister template. To date, it is unclear how the suppression of HR in G1 occurs to prevent centromere recombination with homologous sequences in other chromosomes or within the same chromatid. Activation of HR relies on the generation of single stranded DNA as the DSB is resected. HR or homology-directed repair (HDR) encompasses different sub-pathways but commonly initiates with DNA resection (strand invasion mediated by RecA (in bacteria) or Rad51 (in eukaryotes) that leads to the formation of a displacement loop (D-loop) to create the Holliday junction). A conservative form of HDR is synthesis-dependent strand annealing (SDSA) [186]. SDSA fills DSBs and inhibits crossing over [187]. Because centromeres actively undergo recombination during the mitotic cell cycle [141,147,188] and short- and long-range recombination events are speculated to drive centromere formation and evolution, HR likely represents an active mode of repair for centromere damage. However, this poses important questions on how faithful recognition of the true sister sequence is accomplished, differentiating the many identical and matching sequences within the same chromatid or across chromosomes. Aberrant recombination would give rise to non-allelic exchanges, as we reviewed previously [140].

There are other forms of DNA damage repair whose mutational signatures have been associated with centromere DNAs. Replication fork failure, regression into so-called chicken foot structures and other stalled/collapsed fork conformations can also produce unusual HR substrates, where resolution of the one-ended DSB can be achieved through activation of break-induced repair (BIR) (Figure 3D) [189], or microhomology-mediated break-induced repair (MMBIR) in case of non-sister templates [190,191]. BIR pathway activation on repetitive sequences can cause an out-of-register invasion and the resolution of the D-loop leads to expansions and/or contractions of repeat arrays [192]. Centromere sequences seem to carry a mutational signature compatible with BIR according to a recent report [183].

As an alternative, circular 3′ ssDNA (single stranded DNA) templates generated at the D-loop lead to the induction of rolling circle replication (RCR) (Figure 3E) which occurs preferentially within inverted repeats arrays, generating concatemers [193]. As a result, DNA repair protein RAD51 homolog 1 (RAD51) plays a central role in processing the HJ loop [194], principally with the aim to inhibit single-strand annealing (SSA), an error-prone mechanism that anneals the homologous DNA sequence at the break without a gap, causing a sequence deletion (Figure 3F) [187,195]. SSA results in loss of DNA where the 25-nucleotide strand annealing is followed only by polymerase filling and intermediate ligation [196,197]. Because many of these repair pathways are error-prone, they induce mutagenesis that may favor the evolution of centromere DNA (Figure 3).

Indeed, Rice [183] assigned a contribution to both BIR and SSA pathways in the plasticity of HORs. Contrary to Smith [146], intermingled alternation of CENP-A-enriched/centric core expansion by the BIR pathway during replication, and the length-eroding SSA pathway during the repair of DSBs have converged to enable the formation of homogenized HORs. The latter repair pathway (SSA) appears quite infrequently in centromeric and pericentromeric regions [183]. The large size of the HORs underscores this expansion [189,198]. Furthermore, there is a corresponding increase in CENP-A with expansion of HOR sequence arrays, which in turn leads to increased CENP-A deposition in the form of a positive feedback loop [199,200].

The aforementioned processes cause amplification, expansion and large-scale remodeling of the genomic landscape at the centromere. However, they must also be intersected by localized mutagenesis, including that which triggers divergence between monomers. In the example of the human centromere, individual monomers of α-satellites share only 50–70% sequence identity between each other, while HOR blocks are nearly-identical. Thus, large-scale processes may be rarer and have operated on a wider timescale than small-scale changes and micro-mutations that may continue to shape and diverge centromeres. Notably, BIR seems sufficient to create mutations within the replicated sequences (around 1000-fold with respect to DNA replication without out-of-register forks [183,201]) and results in both long and short-range changes.

A supplementary mechanism to accomplish concomitant mutagenizing and homogenizing of the centromeric repeats is based on inter-chromosomal translocations guided by the organization and proximity of spatial repeats. A high percentage of translocation events has been demonstrated in centromeric homology inverted repeats (HIRs) of common progenitors of *C. albicans* and *C. tropicalis*, in which the loss of these inverted repeats provokes the formation of a new centromere. When the essential function of centromeric HIRs is missing, a CENP-A-rich zone influences the seeding of evolutionary new centromeres (ENCs) in order to reestablish the eroded centromere region [202]. The plasticity of the centromere in establishing into a completely new location adds another layer of complexity in tracking sequence generation through mutagenic processes, where sequences may be originating from diverse and changing ancestral seeding DNA. Yet, these fitting simulations represent important points of reflection to gain a more profound and complete appreciation of the complexity in sustaining centromere evolution and maintenance. Much needed empirical evidence will uncover which of these processes operate within the repetitive satellites through current sequencing efforts. Because mechanisms to suppress processes like HR are emerging [141,147], mutagenic processes, along with their mitigating pathways, will reveal how centromere DNA stability and evolution are maintained.

### Formation of Human Centromeres through Evolutionary Mutagenesis

The DNA organization at human centromeres is a notable example of repeat amplification, homogenization and mutagenesis. One of the first studies on the evolution of human satellite DNA was advanced by Smith in 1976 [153], with the unequal sister crossover model used to describe the dynamic mutability shown by α-satellite repeats. The model explains that the diverse nature of these repetitive sequences is driven by the proportion between the rate of recombination of the mitotic sister chromatid (r), the rate of the base pair mutation (u), and the minimum match length (m) required for unequal crossover [203,204].

More recent advances in methodologies and sequencing allowed the construction of centromere phylogenies to compare centromeres among different organisms, as well as between the same species. Intra- and inter-species analyses are a very helpful tool for the recognition of ancestral and new properties of centromere repeats, exposing evolutionary constrains and adaptive changes over different timescales [201]. In fact, even if the base substitution rate between chimps and human species is only 1.2% in non-centromeric regions (whether or not there is over-repeated and non-repeated DNA [205]), there is a continuous rapid divergence that has been demonstrated through the hybridization of human centromeric DNA probes on the ortholog chimp centromere sequences, suggesting that centromeres have higher degree of divergence [206,207,208]. α-satellite DNA has been found in Old World Monkeys [209,210,211], in New World Monkeys [212,213] and in prosimians [214,215], where it maintains a monomeric, more disordered α-satellite organization [216,217,218]. Instead, α-satellite higher order structure (as found in human centromeres) is also present in our relative Great Apes such as chimpanzees, gorillas [218,219], and orangutans [218,220]. This may reflect a very recent evolution of monomeric satellites into an upper level organization through homogenized HORs [221]. This is particularly interesting as pericentromeres retain monomeric, seemingly ancestral, α-satellite DNA interspersed with Long interspersed nuclear elements (LINEs), Short interspersed nuclear elements (SINEs) and other repetitive elements, suggesting that monomeric α-satellites served as an early template for the HOR homogenization that followed. 

Alexandrov and colleagues advanced a very interesting model about the formation of HOR in Great Apes from an old ancestral monomer in lower primates [209]. Supposedly, the divergence of old monomers prior to the split among human, chimpanzee and gorilla gave rise to a monomer type able to bind CENP-B, creating three supra-chromosomal families (SF) in which both the old and new monomers are alternated [222,223]. In Great Apes, the new type of monomer is present in all chromosomes with some exceptions (e.g., the Y chromosome in humans), although these peculiar cases also have condensed structural organization [224]. In this model, HOR expansion and homogenization could be raised by two different mechanisms: improper replication with the creation of multiple copies (such as rolling circle replication, Figure 3E) [225] and unequal crossovers/gene conversion events (Figure 3A,B) ([154] and [226], respectively). Given the shared layers of α-satellites between chromosomes, it is possible that the newest-born centromere within an old centromere promotes the sliding to the side of the old monomers [227]. New FS arrays, homogenized in chromosome-specific HORs, may facilitate the maintenance of higher order structure through the concomitant recruitment of DNA binding proteins [228]. The integration of the CENP-B box within the HOR array could facilitate kinetochore assembly, yet its absence from the Y chromosome remains unclear [229]. The kinetochore-associated recombination machine (KARM) is proposed to have a role in homogenizing functional centromeres through topoisomerase II-induced breaks that are subsequently repaired by recombination [227].

While evolutionary processes underlying centromere divergence remain unclear [7], a new attractive model was recently provided by Rice [183] by assigning a contribution to all cellular processes involved in the plasticity of HORs, as if HORs have their own molecularly encoded life cycle. The steady drafting of HOR array extension and organization promotes a continued expansion, rather than shrinkage, to generate megabases of homogenized HORs, while SSA contributes to diversity between the individual units [183]. For the longest centromere, the overall size can reach up to 8 Mb [230]. This rapid increment in HOR size cannot be justified solely through antiparallel and unbalanced exchanges between sister chromatids, first due to the exceptional variation found in sex chromosomes and second due to the conserved head-to-tail orientation in all centromeric HORs. Their homogenization seems principally due to replication-associated repair processes that contribute to length diversification and homogenization of the HOR array [183].

The model’s structural frame is based on the spatial organization of three types of ~170 bp monomeric repeat units [231,232] that are predicted to influence centromere strength (i.e., the level of outer kinetochore proteins): (1) one with a protein-binding sequence at its 5′ end (the 17 bp b-box that binds CENP-B), (2) a second that is identical to the first except that the CENP-B-box is mutated so that it no longer binds CENP-B, and (3) a third lacking CENP-B docking site altogether [193].

Among these three monomeric repetitive units, intra-array competition exists. It is based on the capability of centromeric core repeats to extend and migrate towards the flanking heterochromatin region, contrasting it. Thus, this new and interesting model highlights the contrasting forces and high level of evolution caused by the amplification (BIR process), shirking (SSA process) and homogenization of HORs [183].

Inside human HORs, the number of monomers ranges from two (as in chromosome 1 [233]) to 34 monomers (as in chromosome Y) [224,234]. The sequence of monomers has up to 35% variability among chromosomes and within the same chromosome [235], indicating that the formation of HOR followed a different mutagenic process than HOR amplification through homogenization. Despite the human HOR on the Y chromosome possessing alphoid DNA sequences, it differs from the other HORs on autosomes and X chromosomes because it lacks CENP-B boxes [235], indicating that CENP-B is not essential for a functional centromere [72,219]. Notably, some younger HORs with more homogenized monomers [236] that have yet to accumulate additional mutations and SNPs are shared among non-homologous autosomes [237], as for the chromosome groups 1, 5, 19-13, 21-14 and 22 [202]. Some of these sequences are regarded as “pan-centromeric” and are often used for the rapid detection of multiple centromeres in different chromosomes. The fact that we can distinguish between younger and older HORs based on mutational burden implies that either: (1) centromeres are exposed to genetic changes at a high rate, or (2) mechanisms that protect centromeres mitigate for these events yet are not fool proof, leading to the progressive accumulation of mutations.

While chromosomes can contain more than one centromere array with its own set of HORs [238], Sullivan and colleagues have highlighted the striking example of metastable epialleles found on chromosome 17, where three contiguous unique Chr17-specific α-satellite HOR arrays (D17Z1, D17Z1-B, and D17Z1-C) are found within the centromeric region, but only one array is active at any given time [239]. This helps to prevent errors in nucleating the kinetochore and segregating chromosomes during cell division. Interestingly, all arrays still have the ability to recruit CENP-A, acting like epialleles. Yet in the majority of individuals across the human population, the active centromere forms on the main array containing less inter-HOR variation [239]. These data indicate that the homogenization of HOR is functionally important to support centromere function [119,154,239]. As the homogenization of HORs relies on replication fork collapse and re-initiation of replication through BIR and SSA repair processes [183,202], there could be a process in place for a continuous HOR life cycle, beginning with the expansion of α-satellite units as monomers, dimers, and multimeric units, up to full HOR amplification.

Even if these processes are valid and important attempts at placing repetitive pieces of a large puzzle together, experimental evidence is needed to validate their action within human centromeres.

## 4. Changing Identity: Pathological Consequences of Rapid Centromere Evolution

Changes in centromere DNA can reasonably induce chromosome segregation errors and result in chromosome instability (CIN) [140,240]. However, evidence for a direct link between changes in centromere DNA and segregation errors are lacking. Recent work points to centromere size not being a determining feature in aneuploidy, while centromere-specific DNA features such as the presence and density of CENP-B boxes plays a more important role in contributing to centromere function in chromosome segregation [241]. Thus, centromere rapid sequence changes, putative mutagenic processes and intrinsic fragility that converge to undermine centromere repeats would reasonably need to be mitigated to prevent functional disruptions. For instance, dramatic erosion of a centromere may no longer support chromosome segregation, although no defined threshold currently exists for an “optimal” centromere length to perform its function, nor for ribosomal DNA (rDNA) or telomeres. Additionally, DNA changes may impact recruitment and retention of CENP-A and disruption of the CENP-B box for CENP-B recruitment and other essential centromeric components. In addition to size and sequence composition, there are other perilous features, like secondary structure and RNA:DNA hybrids (R-loops) that may need addressing to maintain centromere stability [140,152]. Interestingly, in addition to representing burdens for replication, single-stranded DNA R-loops facilitate homologous sequence matching for BIR crossover [242] that eventually result like gross chromosomal rearrangements (GCRs) [243], a series of processes that may be happening at centromeres.

The pericentromere region is responsible for sister chromatid cohesion and has been found to contribute to centromere integrity [240]. Except for the pericentromeric region of *S. cerevisiae*, where there is a cohesin enrichment [244,245], the pericentromere sequences of fission yeast and other organisms possess heterochromatic characteristics such as high methylation, H3 Lys 9 methylation, cohesin enrichment and the presence of heterochromatin protein 1 (HP1) [246]. Heterochromatin condensation inhibits Transcription elongation factor S-II (Tfs1)-promoted transcription, preventing deleterious transcription–replication conflicts, R-loops and centromere rearrangements [243]. This is reminiscent of mouse centromere recombination being suppressed by the DNA methyltransferases 3 α and β (Dnmt3a/b) that contribute to heterochromatin silencing [147], yet it remains unclear how the cross-talk between the centromere and pericentromere occurs. Hypo-acetylation of centromeric repeats, due to loss-of-function of the de novo methyltransferase DNMT3b, increases DSBs following nucleotide excision repair (NER) [247]. Hypo-acetylation of centromeric repeats, due to loss-of-function of de novo methyltransferase DNMT3b, also increases DSBs caused by NER, which specializes in removing R-loop structures. Mutations in DNMT3b are associated with human immunodeficiency–centromeric instability–facial anomalies (ICF) syndrome [248], as well as mutations in other genes such as CDCA7 [249,250,251], HELLS [249,250,251] and ZBTB24 [252]. DNA hypomethylation of the pericentric heterochromatin of chromosomes 1, 9 and 16 gives rise to peculiar stretched centromeres and chromosome instability in ICF patients [253]. Collectively, this evidence suggests a relationship between transcription, R-loops and other epigenetic features that could either facilitate or undermine the maintenance of centromere stability. Centromere epigenetics may directly influence sequence stability, similarly to how a delicate balance between methylation and acetylation aids CENP-A loading within the centromeric domain [254]. Epigenetic state, nucleosome histone dynamics and changes in specific post-translational modifications (PTMs) impact centromere stability. Because loss of CENPs, especially CENP-A, CENP-C and CENP-T/W, triggers centromere rearrangements [141], maintenance of the epigenetic and proteinaceous components of centromeres is a key component in the stability of DNA repeats. Notably, the proper localization and methylation of CENP-A is essential for cell growth and for the prevention of chromosome instability, together with p53 [255]. Notably, centromere α-satellite stability is compromised in cancer cell lines and in primary cells undergoing senescence [141].

From recent evidence, a shift in CENP-A localization or its depletion led to a change in chromatin status that could interfere with local and long-range transcription processes [243]. This was seen during cellular senescence and leads to CENP-A mislocalization and mitotic arrest [255] in aging cells [256,257,258,259], in cells overexpressing Myc proto-oncogene protein (MYC), under an ectopic interaction with CENP-A [260], and with a corresponding de-repression of centromeric TEs (frequently observed in many cancers) [261,262]. The aberrant transcription of retrotransposons in pericentromeric human satellite II (HSATII) repeats leads to an increased accumulation of centromere RNAs [263], often seen in cancer [169,264,265,266,267]. Thus, temporary and spatial control of transcription may limit the emergence of breaks at centromeres [267,268]. Seemingly, replication timing of the centromere region may be evolutionarily set for spatial purposes [228]. In accordance with the DuPraw’s model [269], the centromere is a late replicating region [92,270] and both the centromere-specific histone H3-like protein CENP-A [271] and CENP-B [272] may contribute to complete the replication process. Yet, the origins, mechanisms and consequences of the replication dynamics at these repetitive regions are poorly understood, and whether replication stress may in turn lead to breakage and, as previously described, trigger catastrophic rearrangements, is unclear [140]. Once a break is generated, fork stalling and template switching (FoSTeS), non-allelic homologous recombination (NAHR), BIR and MMBIR pathways may repair the chromosomal break and produce unbalanced translocations, isochromosomes, acentric chromosomes generating fragment loss, ring chromosomes, dicentric chromosomes, Robertsonian translocations, pseudo-dicentric chromosomes and other gross chromosomal rearrangements, leading to aneuploidy (for further information, see [140,240]). Because these genomic aberrations represent a potential source of instability with numerical and structural alterations found in multiple cancers [240], fully understanding their molecular origins is of great importance.

## 5. Conclusions

Centromeres hold multiple paradoxes, including rapidly evolving DNA and molecular players, tolerance to changes in position and size, evidence of profound mutational processes, intrinsic fragility dictated by repetitive DNA and possibly secondary structures, all while maintaining a fundamental and conserved function. Interestingly, centromere sequences reveal the preferential accumulation of tandem repeats and a conserved epigenetic identity as the driving force for maintaining centromere function in spite of their high mutational rate. Such molecular processes affect the clusterization of satellite DNA and its higher order assembly, but it is not exactly clear at which level they operate and especially what the mechanisms are that preserve centromere DNA stability and mitigate ongoing mutagenesis. Because of its genetic variability, the unifying definition of a centromere refers to its functionality in enabling chromosome segregation. Under evolutionary forces, each organism modulated this essential structure based on their evolutionarily benefits, mitigating drawbacks and tolerating adaptation. Thus, the changes in DNA content, size and positional shifts, as well as the three-dimensional arrangements of increasing complexity that can converge to sustain a loop of mutagenesis that feeds centromere evolution, are both an advantage or a hindrance depending on the timescale snapshot (within a few generations or selected over millions of years). Looking at centromere abnormalities widely found in cancer and other disorders, the precarious equilibrium between rapid changes and functional preservation in the quest for the sustained propagation of centromeres likely comes at a cost in conflict with DNA stability. We do not yet know the complete journey that makes a centromere so, but it is certainly an exciting and eventful one that we hope will soon fully emerge.

## Figures and Tables

**Table 1:** Centromere structure in different species.

Centromere Type	Species	Size	References
Point centromere	**fungi**		[4]
	*Saccharomyces cerevisiae*	~125 bp	
Short regional centromere	**fungi**		[9,10]
	*Candida albicans*	~3–5 kb	
	*Schizosaccharomyces pombe*	~35–110 kb	
Long regional centromere	**viridiplantae**		[11,12,13]
	*Arabidopsis thaliana*	~400 kb–1.4 Mb	
	*Oryza sativa*	~65 kb–2 Mb	
	*Zea mays*	~180 kb	
	**metazoa**		[14,15,16]
	*Drosophila melanogaster*	~420 kb	
	*Mus musculus*	~1 Mb	
	*Homo sapiens*	~0.5 to 5 Mb	
Meta-polycentric centromere	**tracheobionta**		[17]
	*Pisum sativum*	~69–107 Mb	
Holocentromere	**viriplantae**		[18]
	*Luzula nivea*	~100 Mb	
	**metazoa**		[19,20,21]
	*Bombyx mori*	~8–21 Mb	
	*Caenorhabditis elegans*	~14–21 Mb	

**Table 2:** H3-like centromeric protein A homologues in different model organisms.

H3-Like Centromeric Protein A Homologues	Model Organism	Size
Chromosome segregation 4 (Cse4)	*Saccharomyces cerevisiae* [28]	~26 kDa [29]
Centromere-specific histone H3 (Cnp1)	*Schizosaccharomyces pombe* [30]	~13 kDa [31]
Centromere identifier (Cid)	*Drosophila melanogaster* [32]	~25 kDa [33]
Centromeric histone 3 (CenH3)	*Arabidopsis thaliana* [34]	~19 kDa [35]
Histone H3-like centromeric protein (HCP-3)	*Caenorhabditis elegans* [36]	~32 kDa [37]
Centromeric protein A (Cenpa)	*Mus musculus* [38]	~15 kDa [39]
Centromeric protein A (CENP-A)	*Homo sapiens* [40,41]	~15 kDa [42]
